# Epigenetically altered miR-1247 functions as a tumor suppressor in pancreatic cancer

**DOI:** 10.18632/oncotarget.15722

**Published:** 2017-02-24

**Authors:** Joo Mi Yi, Eun-Jin Kang, Hyun-Mi Kwon, Jin-Han Bae, Keunsoo Kang, Nita Ahuja, Kwangmo Yang

**Affiliations:** ^1^ Research Center, Dongnam Institute of Radiological and Medical Sciences (DIRAMS), Busan, Republic of Korea; ^2^ Department of Microbiology, Dankook University, Cheonan, Korea, Republic of Korea; ^3^ Department of Surgery, Oncology, and Urology, The Johns Hopkins University School of Medicine, Baltimore, Maryland, USA

**Keywords:** microRNA, miR-1247, hypermethylation, tumor suppressor, pancreatic cancer

## Abstract

Altered expression of microRNAs has been strongly implicated in human cancers, and growing evidence is emerging that a number of miRNAs are downregulated in cancer associated with CpG island hypermethylation. Although pancreatic cancer is one of the most malignant human cancers, the roles of miRNAs underlying the tumorigenesis of pancreatic cancer are still poorly understood. In the present study, we explored the molecular functional role of microRNA-1247 as tumor suppressor associated with epigenetic alteration in pancreatic cancer. CpG islands methylation of miR-1247 is frequently observed in various pancreatic cancer cell lines and in primary pancreatic tumors, but not in normal pancreatic tissue. Ectopic expression of miR-1247 in five pancreatic cancer cell lines results in suppressing of cell growth, proliferation, migration, and invasion *in vitro* and tumorigenicity of pancreatic cancer cells *in vivo*. Interestingly, we found one putative target gene of miR-1247, regulator of chromosome condensation 2 (*RCC2*), harbored miR-1247 target sequences in the 3′ UTR of its mRNA. In functional studies *in vitro* to understand the interaction between miR-1247 and *RCC2*, decreasing of *RCC2* gene expression by miR-1247 was observed by immunoblotting and immunohistochemistry at both mRNA and protein levels. Moreover, luciferase reporter assay confirmed that *RCC2* was a direct target of miR-1247. Taken together, our data suggest that CpG island hypermethylation of miR-1247 is responsible for its downregulation in pancreatic cancer, and ectopic expression of miR-1247 functions as a potential tumor suppressor targeting *RCC2* in pancreatic cancer cells.

## INTRODUCTION

Pancreatic cancer is one of the most aggressive malignancies. During the past decades, although great efforts have been made to improve early detection and clinical outcomes of patients with pancreatic cancer [1], the overall prognosis remains poor, with a 5-year survival rate of ∼8% [2]. Therefore, it is critical to understand the molecular mechanisms underlying progression of pancreatic cancer and elucidate signaling pathways that might serve as cancer treatment targets.

MicroRNAs (miRNAs) are small noncoding RNA molecules, 18–25 nucleotides in length, and it has been well characterized that they play critical functions across biological processes, including cell proliferation, differentiation, and apoptosis [3, 4]. Emerging evidence from miRNA expression profiling has revealed that most miRNAs are expressed at lower levels in tumors than in normal tissues, whereas some miRNAs are upregulated or unchanged [5]. Therefore, numerous studies have shown that some miRNAs are downregulated in various cancers, indicating that they may act as tumor suppressors [6–8]. For example, the family of let-7 miRNAs represents clear tumor suppressor miRNAs that consist of a group of highly conserved miRNAs across species including *Caenorhabditis elegans*, *Drosophila*, and vertebrates. *Let-7* is generally expressed at low levels in human cancer, and the *Ras* oncogene has been demonstrated to be directly regulated by human *let-7* [9].

Although the mechanisms underlying miRNA dysregulation in cancer are not yet fully understood, recent studies have shown that silencing of several miRNAs is tightly linked to epigenetic mechanisms, such as DNA methylation and histone modification. With screening of epigenetically silenced miRNAs in many cancer cells, the list of miRNA genes methylated in cancer is rapidly growing [10–12]. Inactivation of tumor suppressor genes in human cancers is due to promoter CpG island hypermethylation which is one of the most common mechanisms [13]. Emerging studies support the idea that dysregulation of miRNA expression in cancer is also mediated by DNA methylation [14]. Therefore, these data lead to identification of tumor suppressor miRNA candidates whose silencing is associated with CpG island hypermethylation. The epigenetic silencing of protein-coding genes is a significant mechanism of downregulating tumor suppressor functions in several tumors [13] and a number of tumor suppressor miRNAs are known to be downregulated by DNA methylation in many cancers [14, 15]. However, little has been reported about miRNAs dysregulated by epigenetic alterations and their functional roles in pancreatic cancer.

In the present study, we found that CpG island in the upstream region of miR-1247 gene was frequently hypermethylated correlated with transcriptional silencing of miR-1247 in pancreatic cancer cell lines and primary tumors. We also determined that ectopic expression of miR-1247 resulted in robust reduction of cell proliferation, migration, and invasion. This suppression of cell growth by miR-1247 was confirmed in a xenograft model. Further functional analyses of miR-1247 revealed that it directly represses the regulator of chromosome condensation 2 (*RCC2*) by targeting 3′untranslating region. Our results provide that miR-1247 is epigenetically regulated by hypermethylation and may function as a tumor suppressor in pancreatic cancer.

## RESULTS

### MiR-1247 is epigenetically silenced by CpG island hypermethylation in pancreatic cancer

**Figure 1:**
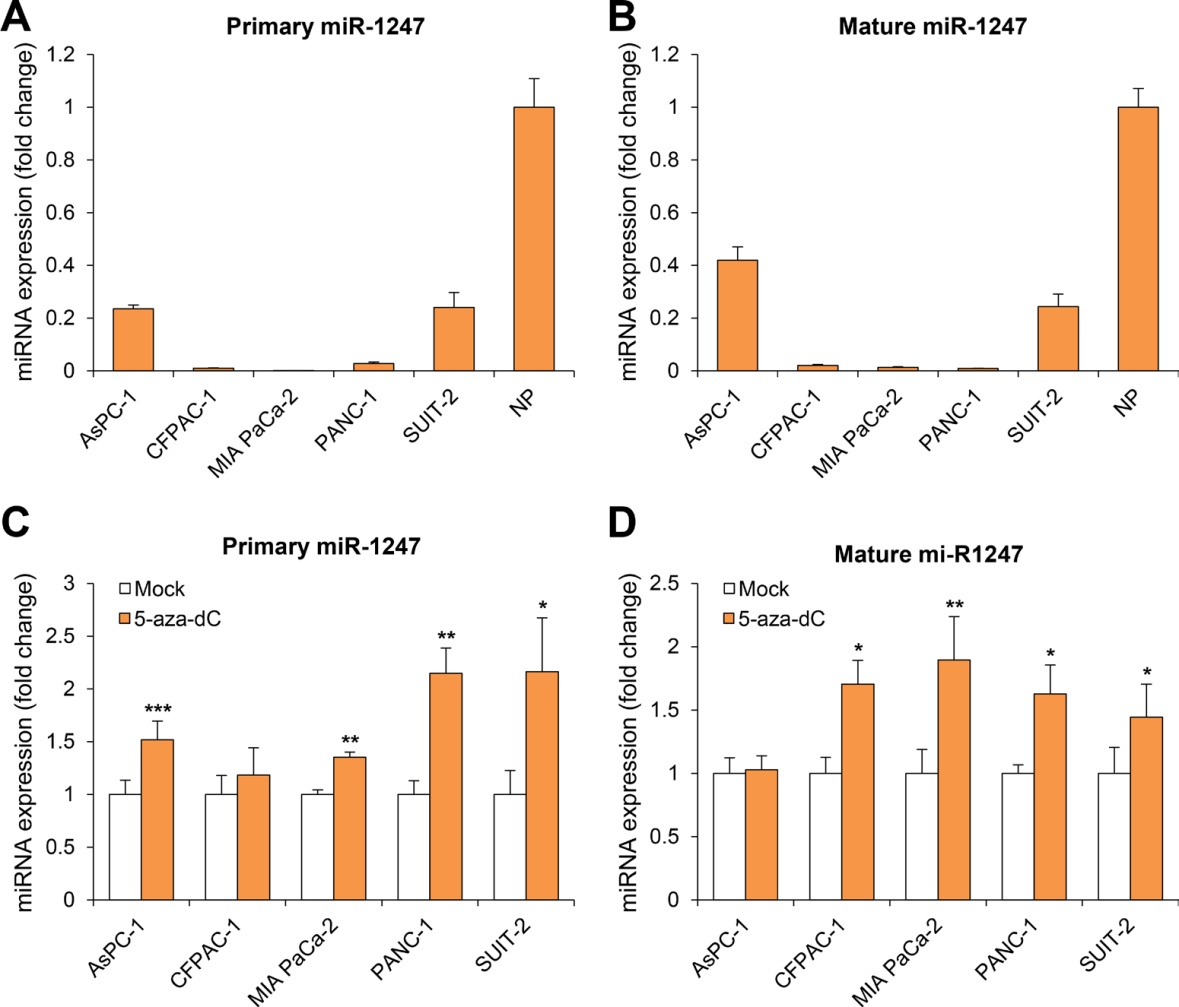
Analysis of miR-1247 expression in pancreatic cancer cell lines (**A**–**B**) Quantitative RT-PCR (qRT-PCR) analysis of expression pattern of pri-miR-1247 and mature miR-1247 in pancreatic cancer cell lines (AsPC-1, CFPAC-1, MIA PaCa-2, PANC-1, and SUIT-2) and normal pancreatic tissue (NP). (**C**–**D**) qRT-PCR analysis of both pri-miR-1247 and mature miR-1247 levels in five pancreatic cancer cell lines before and after treatment with 5 μM 5-aza-dC for 72 h. Dark gray bar indicates 5-aza-dC treatments. *indicates significant increase in pri-miRNA and mature miRNA expression after 5-aza-dC treatment. Data are presented as mean ± SD from one of three independent experiments performed. *P < 0.05, **P < 0.01, ***P < 0.001.

Previous report had been demonstrated that miR-1247 is regulated by DNA hypermethylation in colon cancer using both gene expression and genome-wide methylation profiles [16]. To investigate whether miR-1247 might be regulated by DNA hypermethylation in pancreatic cancer, we first analyzed the expression of miR-1247 in five pancreatic cancer cell lines and normal pancreatic tissue using quantitative RT-PCR (qRT-PCR). Both primary and mature miR-1247 are indeed transcriptionally downregulated in most pancreatic cancer cell lines compared to expression level of normal tissue (< 5-fold) (Figure 1A, 1B). We wondered whether its transcriptional downregulation of miR-1247 is under epigenetic control, and so we treated the demethylating agent 5-aza-2′-deoxycytidine (5-aza-dC) to five pancreatic cancer cell lines (AsPC-1, CFPAC-1, MIA PaCa-2, PANC-1, and SUIT-2) and performed qRT-PCR analysis of both pri-miR-1247 and mature miR-1247. We observed significantly increased expression of pri-miR-1247 after 5-aza-dC treatment in most pancreatic cancer cell lines, but not in CFPAC-1 cells (Figure 1C). We also examined whether the expression of mature miRNAs could be restored by 5-aza-dC in pancreatic cancer cell lines (Figure 1D). We confirmed that mature miR-1247 was re-expressed after treatment with 5-aza-dC, consistent with studies in most other pancreatic cancer cell lines (however, not in AsPC-1 cells) that measured primary transcript of miR-1247. Considering the basal expression level and re-expression level after 5-aza-dC, we suspect that CpG islands in the promoter region of miR-1247 are hypermethylated in pancreatic cancer cells. We therefore asked whether DNA methylation in the promoter region of miR-1247 is responsible for the transcriptional silencing of miR-1247 in pancreatic cancer cells.

### Correlation between methylation and transcriptional expression of miR-1247 in pancreatic cancer cells and primary tumors

**Figure 2:**
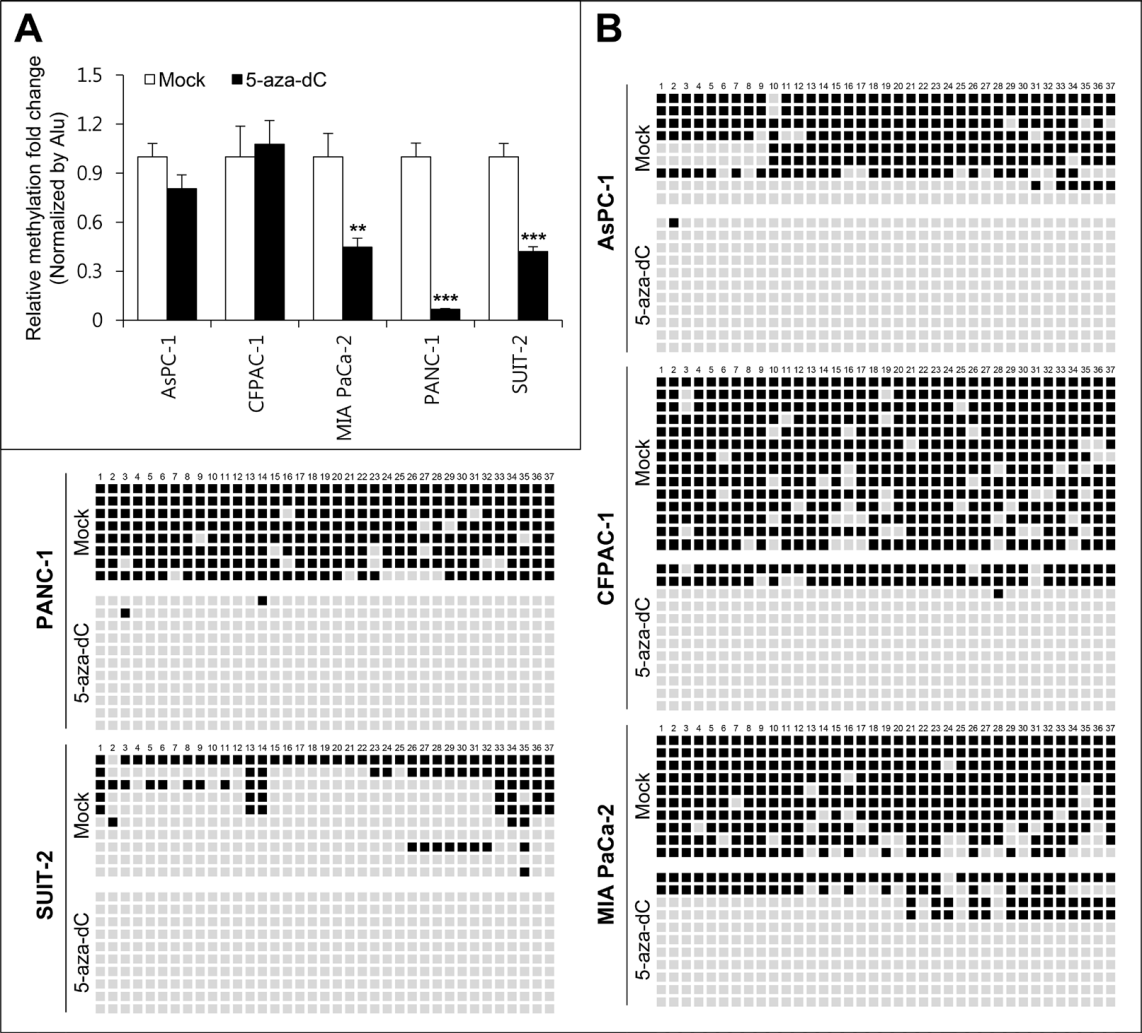
Methylation analyses of miR-1247 in pancreatic cancer cell lines (**A**) Quantitative methylation-specific PCR (qMSP) analysis of methylation level of miR-1247 in pancreatic cancer cell lines. All quantitated methylation levels were normalized to the *Alu* element. Statistical significance (**P < 0.01, ***P < 0.001) is shown between mock- and 5-aza-dC-treated samples for three separate cell lines. (**B**) Representative results bisulfite sequence analysis of miR-1247 in pancreatic cancer cell lines. Bisulfite sequence analysis was carried out for miR-1247 in AsPC-1, CFPAC-1, MIA PaCa-2, PANC-1, and SUIT-2 cells. Black represents methylated CpG sites whereas white represents unmethylated CpG sites.

**Figure 3:**
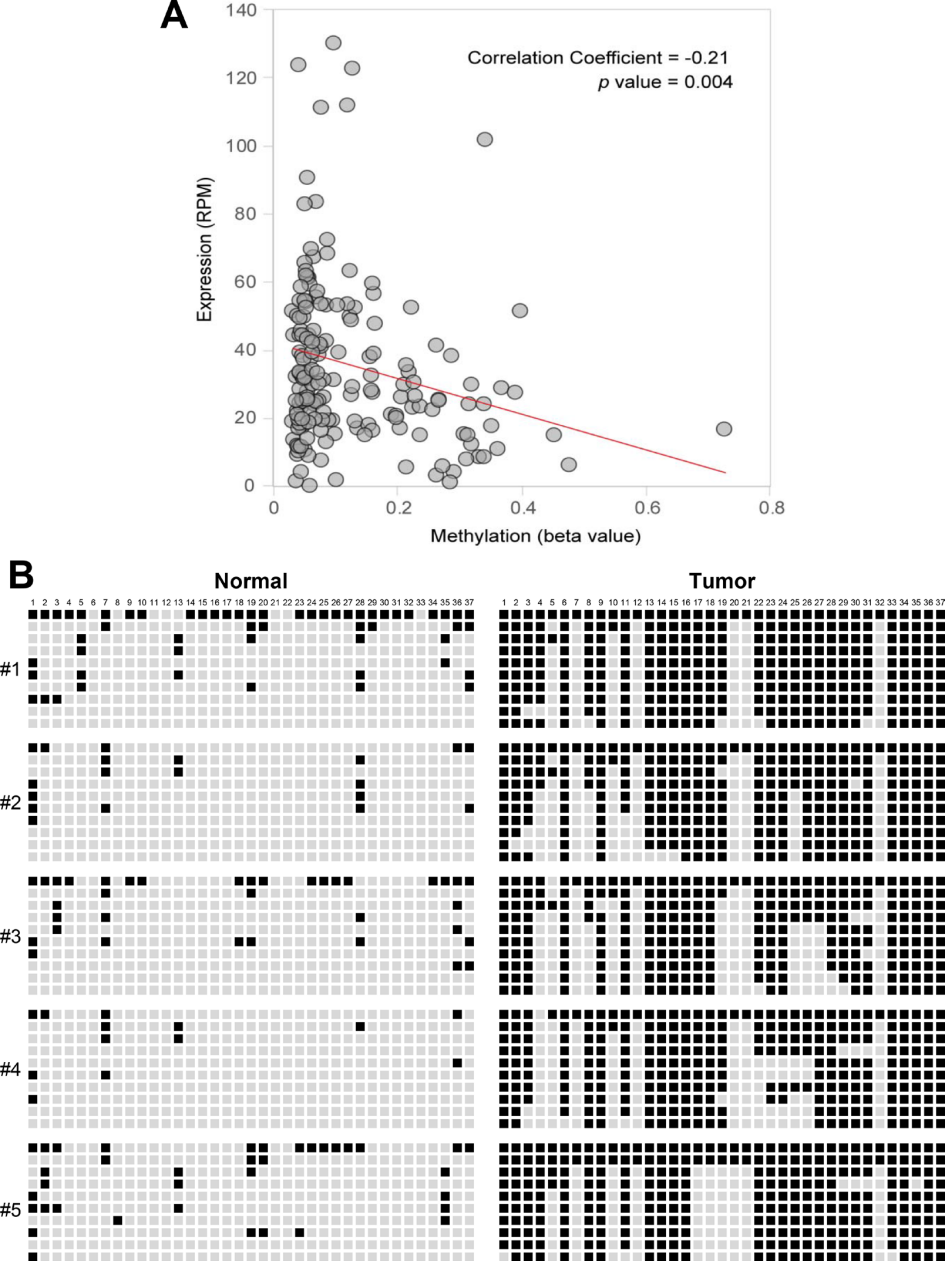
Correlation between expression levels and methylation beta-values of miR-1247 in pancreatic primary tissue samples (**A**) Pearson's correlation coefficient was used to determine the correlation between the methylation level (beta value; x axis) of miR-1247 locus and the expression level (RPM, reads per million; y axis) of miR-1247 in 177 tumor samples. The data were obtained from the TCGA database. (**B**) Bisulfite sequencing was carried out for miR-1247 in five cancer-free normal samples and five primary pancreatic tumor tissues. Bisulfite sequenced regions are indicated with genomic coordinates from Hg19 (UCSC Genome browser). Sequencing region is located at 101,095,801∼101,096,127 (326 bp) in chromosome 14. Each box represents a CpG dinucleotide. Black boxes represent methylated cytosines while white boxes represent unmethylated cytosines. Values for Venn diagram represent number of methylated CpGs relative to total CpGs per allele for cell lines. Black represents methylated CpG sites, whereas white represents unmethylated CpG sites.

Quantitative methylation-specific PCR (qMSP) analysis was performed to assess the methylation pattern of CpG islands upstream of miR-1247 gene locus in pancreatic cancer cell lines and in 5-aza-dC treated cells. We observed that CpG island was methylated in most of the pancreatic cancer cell lines we tested, and MIA PaCa-2, PANC-1, and SUIT-2 cells showed demethylation with 5-aza-dC treatment (Figure 2A). To confirm this, we assessed DNA methylation level in the proximal region of miR-1247 by bisulfite sequencing analysis (Figure 2B). MiR-1247 showed dense DNA methylation in pancreatic cancer cells and demethylation (76%–100%) was observed in cells upon 5-aza-dC treatment, strongly supporting idea that the re-expression of silenced miR-1247 by 5-aza-dC was leading to DNA demethylation (Figure 2). Therefore, we wonder whether methylation of miR-1247 is correlated with its expression in pancreatic tumors by analyzing data set of pancreatic cancer samples (*n* = 177) from the Cancer Genome Atlas (TCGA). Investigation of methylation and transcriptional expression in the large TCGA dataset revealed that methylation and transcriptional expression of miR-1247 were inversely correlated (correlation coefficient = −0.21, *p* = 0.004) (Figure 3A). Therefore, we concluded that miR-1247 is definitely epigenetically silenced in pancreatic cancer cells. We next analyzed the methylation status in pancreatic cancer and normal pancreatic tissue specimens by bisulfite sequencing analysis (Figure 3B). We observed significant dense methylation level of miR-1247 in pancreatic cancer samples compared with normal tissue. These data suggest that hypermethylation in the proximal region of miR-1247 has a cancer specific manner correlated with aberrant transcriptional silencing of miR-1247 in primary pancreatic tumors.

### Functional effects of miR-1247 overexpression in pancreatic cancer cells

**Figure 4:**
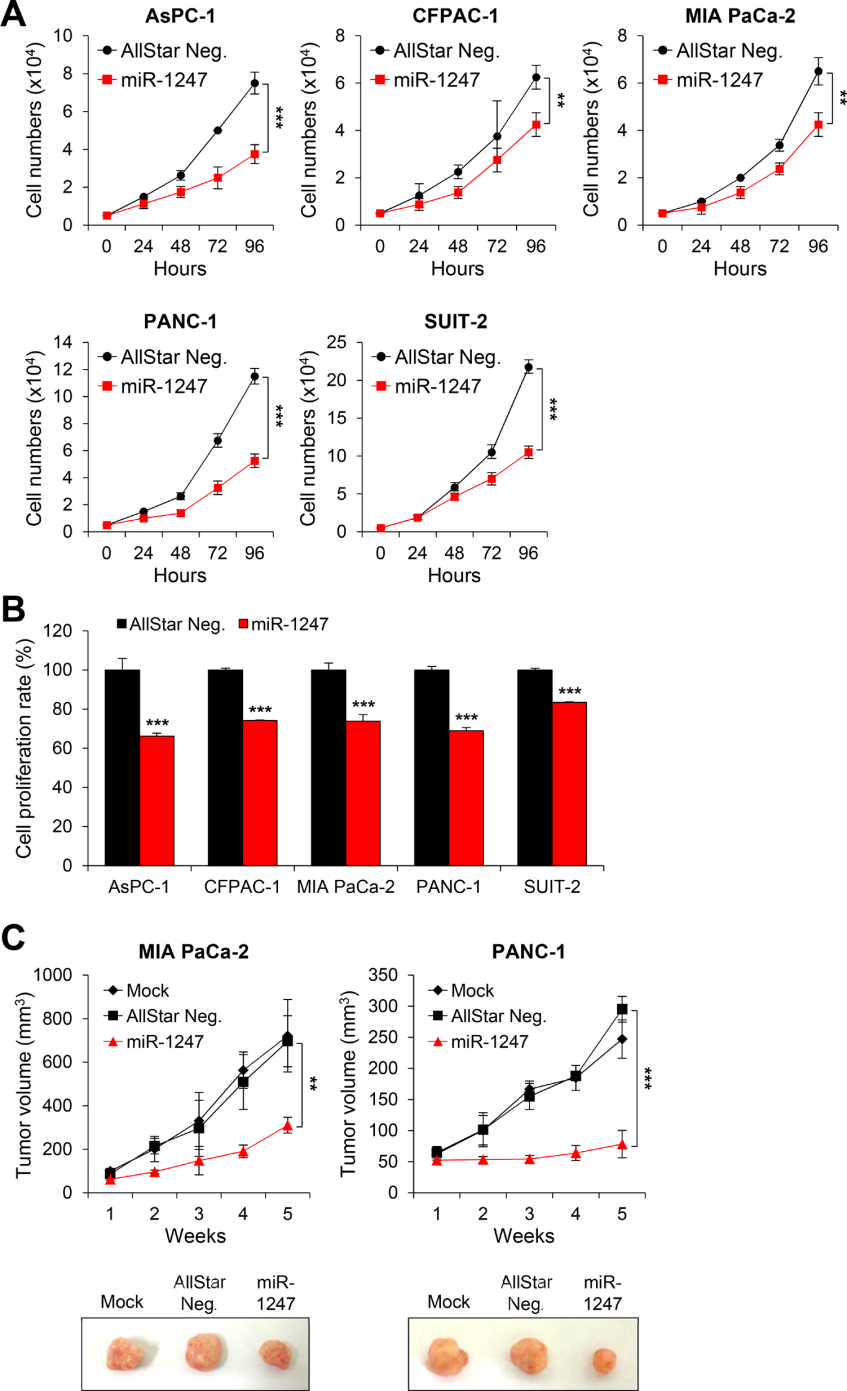
Overexpression of miR-1247 in pancreatic cancer cells inhibits cell growth and proliferation *in vitro* and *in vivo* (**A**) Growth curves and (**B**) MTT assays of pancreatic cancer cells (AsPC-1, CFPAC-1, MIA PaCa-2, PANC-1, and SUIT-2) transfected with non-coding negative control (AllStar Neg.) or miR-1247 mimics. (**C**) Nude mice xenograft model. MiR-1247 suppressed *in vivo* tumor growth. The three groups included mock (*n* = 5), transfection of non-coding negative control (AllStar Neg.) (*n* = 5) and transfection of miR-1247 (*n* = 5). Tumor volumes were measured at the indicated time points after tumor cell inoculation. *indicates statistically significant decrease in cell growth compared to control (*P < 0.05, **P < 0.01, ***P < 0.001).

To determine whether epigenetically regulated miR-1247 has tumor-suppressive properties in pancreatic cancer cells, we transfected five pancreatic cancer cell lines with miRNA mimics of miR-1247 and non-coding negative controls. We first carried out cell growth-curve analysis and MTT assay (Figure 4) to assess cell growth and cell viability, respectively. We found that 72 h after transfection, ectopic expression of miR-1247 suppressed pancreatic cancer cell growth (Figure 4A). Cell growth was inhibited by greater than 40% in most cell lines at 72 h (Figure 4A). All five pancreatic cancer cell lines showed 20%–40% decrease in fitness with the MTT assay readout compared with controls (Figure 4B). Based on *in vitro* data showing significant growth inhibition in cells expressing ectopic miR-1247, we investigated whether miR-1247 could suppress pancreatic cancer tumorigenicity *in vivo*. MIA PaCa-2 and PANC-1 cells (5 × 10^6^) were subcutaneously injected into immunocompromised nude mice. After 5 weeks, the tumors were harvested and it was noteworthy that tumors formed in the miR-1247 group were much smaller than those in the group transfected with non-targeting negative control (Figure 4C). In agreement with the cell growth curve, the volumes of tumors treated with miR-1247 mimics were significantly smaller than tumors injected with non-coding negative control.

**Figure 5:**
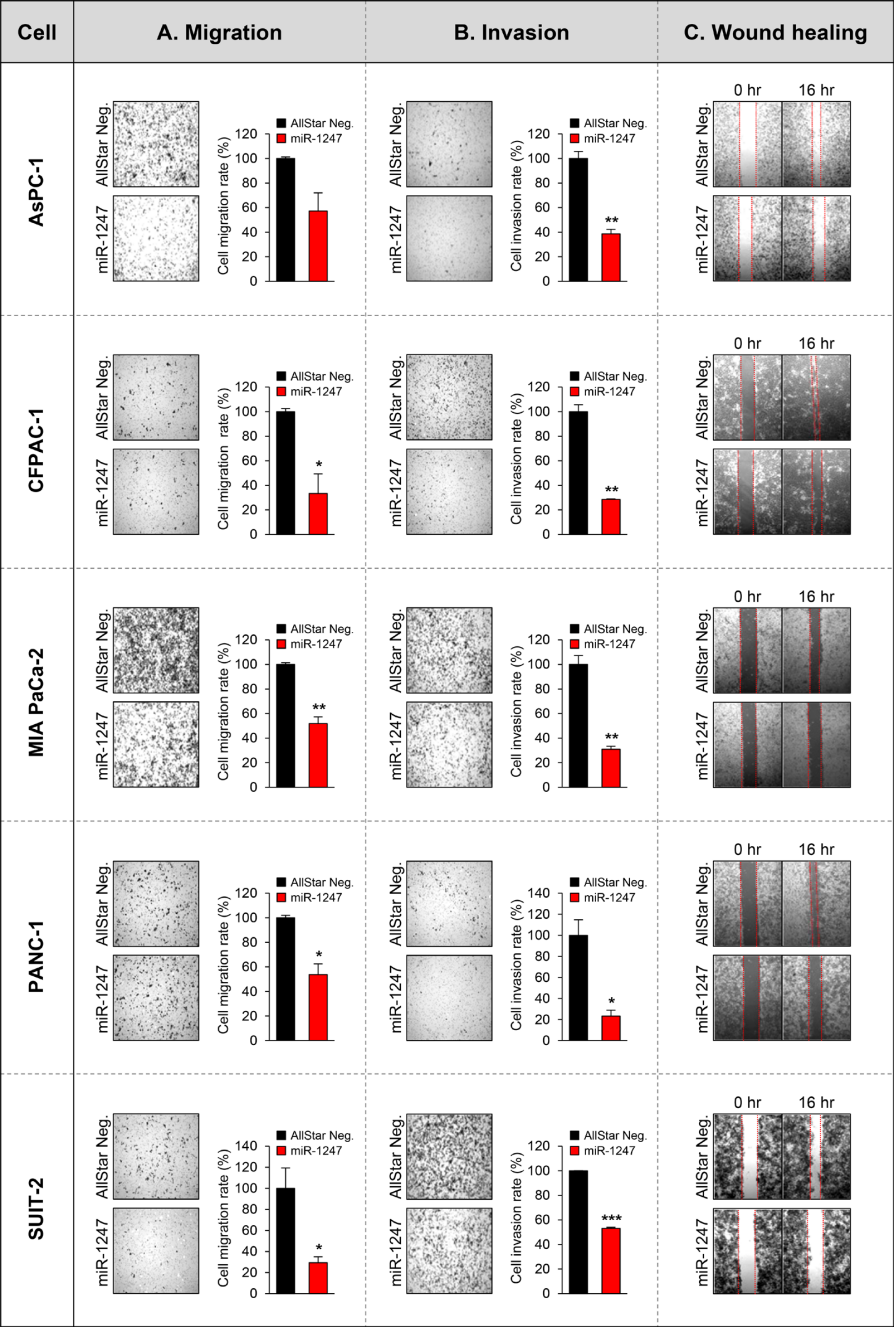
Functional analyses of miR-1247 in pancreatic cancer cells (**A**) The bar graph represents the quantity of migrated cells in transwell migration assay of pancreatic cancer cells transfected with non-coding negative control (AllStar Neg.) or miR-1247 mimics. Quantity of migrated cells represents the mean of three randomly selected microscopic fields per membrane and error bars represent SD. (**B**) Transwell invasion assay using pancreatic cancer cells transfected with non-coding negative control (AllStar Neg.) or miR-1247 mimics. A representative field of invasive cells on the membrane is shown. The data represent the mean of three randomly selected microscopic fields per membrane, and error bars represent SD. (**C**) Wound-healing assay of indicated pancreatic cancer cells mock-transfected or transfected with non-targeting negative control (AllStar Neg.) or miR-1247 mimics. Photographs were taken 16 h after wounding. *indicates statistically significant decrease in migration and invasive ability compared to control (*P < 0.05, **P < 0.01, ***P < 0.001).

These data suggest that ectopic expression of miR-1247 significantly decreased growth and metabolism of pancreatic cancer cell lines. Furthermore, we performed migration and invasion assays to analyze the effect of miR-1247 expression on pancreatic cancer cell migration and invasion. We found that pancreatic cancer cells expressing ectopic miR-1247 suppress cell migration and invasion. Interestingly, we observed significant inhibition of migration (ranging from 42% to 70%) and invasion (ranging from 70% to 87%) with miR-1247 expression in all pancreatic cancer cell lines we tested (Figure 5A, 5B). In agreement with these results, wound-healing assay showed that pancreatic cancer cells (AsPC-1, CFPAC-1 MIA PaCa-2, PANC-1, and SUIT-2) we tested transfected with miR-1247 mimics were significantly impaired in their ability to repopulate wounded areas when compared to non-coding negative control. On the other hand, miR-1247 mimic transfected cells migrated into the wound at a much slower rate in most pancreatic cancer cells (Figure 5C). Overall, these results strongly suggest that miR-1247 has tumor suppressor functions in pancreatic cancer cells both *in vitro* and *in vivo*.

### *RCC2* is a target of miR-1247 in pancreatic cancer cells

**Figure 6:**
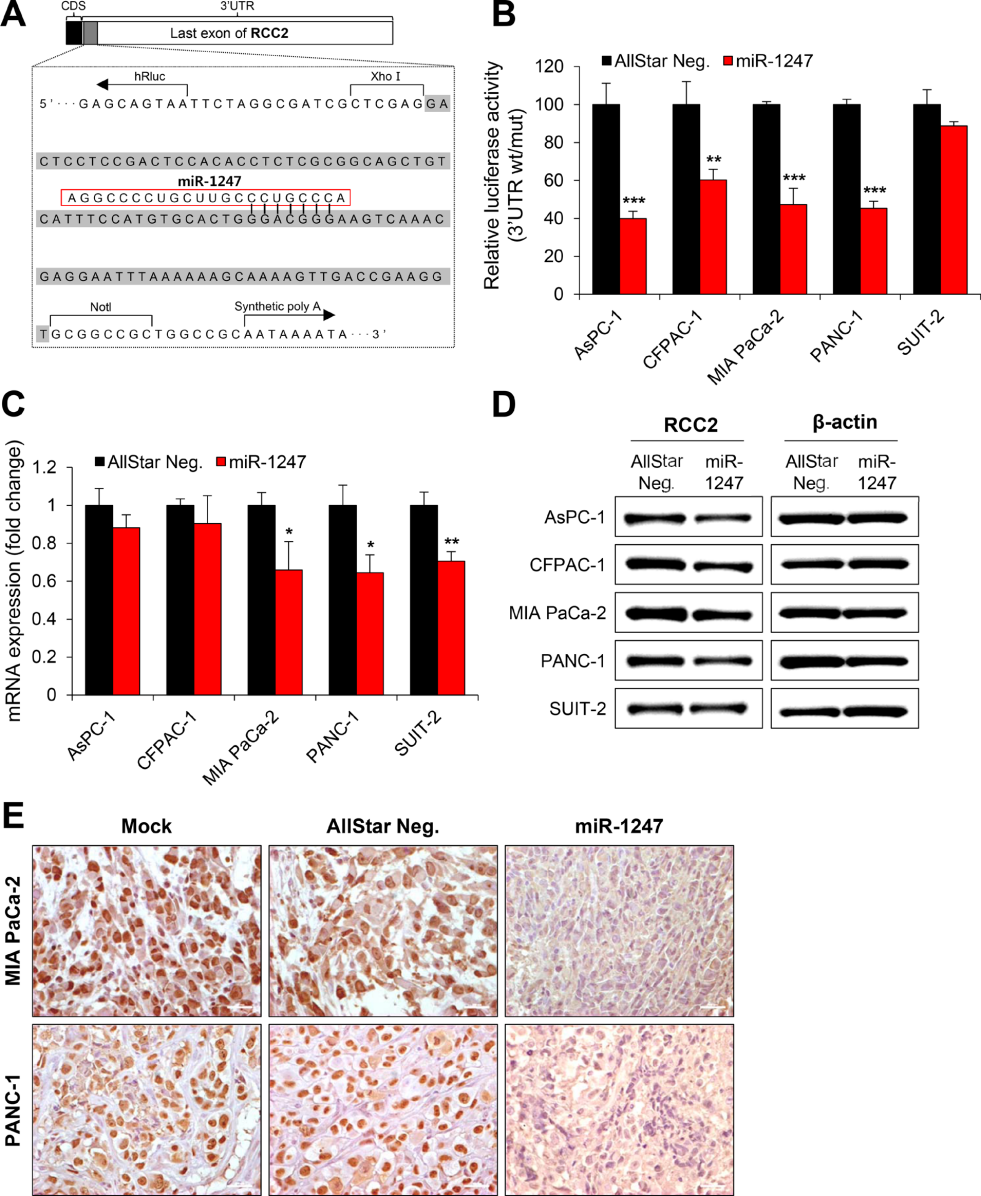
MiR-1247 targets RCC2 in pancreatic cancer cells (**A**) Schematic diagram of RCC2 3′ UTR region indicating putative miR-1247 binding sites. Sequences in the gray bar were cloned into the psiCHECK2 vector for luciferase assay. (**B**) Relative luciferase activity of the indicated RCC2 reporter construct in five pancreatic cancer cell lines co-transfected with miR-1247 mimics or non-targeting negative controls (AllStar Neg.) is shown. (**C** and **D**) qRT-PCR (C) and Western blotting (D) were performed to detect expression of RCC2 after transfection with miR-1247 mimics or non-targeting negative control (AllStar Neg.). * indicates statistically significant decrease in luciferase activity and gene expression level compared to control (*P < 0.05, **P < 0.01, ***P < 0.001). (**E**) Immunohistochemical analysis of sections from tumors of the mice injected with MIA PaCa-2 and PANC-1 cells transfected with negative control (AllStar Neg.) or miR-1247 mimics.

We next focused on the mechanism underlying the tumor suppressor function of miR-1247 in association with its target genes. We previously identified several putative target genes of miR-1247 by target prediction algorithm “TargetScan” in colorectal cancer [17]. We therefore screened 13 genes (*BLCAP, BRSK1, CPEB4, CREBZF, IGSF9B, MOCS1, MYCBP2*, *PHF21A, RARA, RBM19, RCC2, STX1B*, and *ZBTB46*) in five pancreatic cancer cell lines to determine whether miR-1247 induce transcriptional changes of candidate genes (Supplementary Figure 1). Interestingly, *RCC2* showed significant transcriptional downregulation due to expression of mimic miR-1247 in MIA PaCa-2, PANC-1, and SUIT-2 cells, whereas *RCC2* mRNA expression was slightly decreased in AsPC-1 and CFPAC-1 cell lines (Supplementary Figure 1). It is well known that miRNAs regulate mRNA expression by targeting the 3′ UTR of mRNAs with a complementary seed sequence. Using a promoter–activity-prediction program, we found putative binding sites of miR-1247 in the 3′ UTR of *RCC2* (Figure 6A). Therefore we examined whether miR-1247 is able to interact directly with the 3′UTR of *RCC2*. The miR-1247 binding sequence at the 3′UTR of *RCC2* mRNA (wt) was cloned downstream of the firefly luciferase reporter gene, and then cotransfected with mimic miR-1247 or AllStar negative control into the pancreatic cancer cell lines. When miR-1247 was cotransfected, the relative luciferase activity of the reporter containing *RCC2* wt-3′UTR was significantly suppressed ranging from 15% to 60% in comparison to AllStar negative control (Figure 6B). These results indicate that miR-1247 is able to regulate the expression of *RCC2* by directly binding to its 3′UTR. In agreement with these results, *RCC2* mRNA and protein levels were also significantly decreased in the pancreatic cancer cell lines overexpressing miR-1247 (Figure 6C, 6D). To make sure this point, immunohistochemical analysis was performed to measure the protein levels of *RCC2* in tumor tissues of nude mouse xenograft model we analyzed in Figure 4C. The data showed that overexpression of miR-1247 mimics decreased *RCC2* expression in the tumor tissues compared to control tissues (Figure 6E). We conclude that overexpression of miR-1247 is responsible for decreasing *RCC2* levels by targeting 3′UTR in pancreatic cancer.

## DISCUSSION

Emerging evidence indicates that miRNAs often act as oncogenes or tumor suppressors to regulate many cellular events in different steps of tumor development and progression [18]. Although a few miRNAs have been well studied in colon and breast cancers, the role and molecular mechanisms of many other miRNAs in pancreatic cancer are not well known. MiR-1247 was first identified in colorectal cancer and reported to be downregulated in colorectal and gastric cancers [16, 17]. We provided in this study as first evidence that epigenetically downregulated miR-1247 could play a role as tumor suppressor in pancreatic cancer.

Dysregulation of miRNA expression is pervasive in many cancers, and epigenetic mechanisms are key mediators of downregulation of tumor suppressor type miRNA expression. MiR-127, miR-9, miR-148, miR-203, miR-340, miR-18, and miR-34b/c are several examples of miRNAs regulated by CpG island hypermethylation [11, 15, 19, 20]. Recently, miR-34b/c has been reported to be epigenetically regulated in several cancers [15, 21]. MiR-34b/c was shown to be a tumor suppressor mediating p53 activation in both colon and gastric cancers [11, 22, 23]. In addition, methylation of miR-124 families was initially described in colorectal cancer and was subsequently reported in tumors of other origins. We then hypothesized that miR-1247 may be frequently hypermethylated in pancreatic cancer, as in the examples above, with significant biological roles associated with tumorigenesis.

Hypermethylation of CpG islands is known to be an epigenetic aberration that leads to inactivation of tumor-suppressive genes in cancer cells [13]. Recent studies clearly addressed transcriptional downregulation mediated by hypermethylation of tumor suppressive miRNA genes in various types of cancers [15]. Here, we found that miR-1247 is frequently downregulated in pancreatic cancer cells compared with normal pancreatic tissue and its expression is restored by 5-aza-dC treatment, suggesting that abnormal expression of miR-1247 is epigenetically regulated due to DNA hypermethylation in pancreatic cancer cells. Based on expression analysis of miR-1247 in pancreatic cancer cell lines treated with 5-aza-dC, we strongly suspected that DNA methylation was one of the regulatory mechanisms of miR-1247 expression. In addition, expression and methylation profiles in pancreatic cancer obtained from TCGA database showed significant inverse correlation which supporting that expression of miR-1247 is regulated by CpG island hypermethylation. In order to confirm the DNA methylation associated with abnormal expression of miR-1247, bisulfite sequencing analysis was performed to assess the methylation status of CpG islands of miR-1247, and we observed that miR-1247 is hypermethylated in five pancreatic cancer cell lines. Correspondingly, CpG islands hypermethylation of miR-1247 was also observed in pancreatic tumor specimens, supporting the idea that epigenetic silencing of miR-1247 is truly regulated by CpG island hypermethylation.

It is well known that miRNAs participate in regulating the biological effects such as cell growth, proliferation, differentiation, and apoptosis, by controlling the expression of their target genes. Our functional analyses described that miR-1247 mimic treated pancreatic cancer cells showed strong inhibitory properties of miR-1247 in cell growth, migration, and invasion, demonstrating that miR-1247 acted as a tumor suppressor *in vitro*. Notably, restoration of miR-1247 clearly inhibited pancreatic tumorigenesis and suppressed tumor growth *in vivo*, strengthening our conclusion that miR-1247 functions as a tumor suppressor in pancreatic cancer. However, the functions, target genes, as well as the regulatory mechanism of miR-1247 are still unknown in pancreatic cancer. In our study, we showed that miR-1247 reduced the expression level of *RCC2* at both mRNA and protein levels in pancreatic cancer cells. The luciferase activity assay with a reporter containing the miR-1247 binding sequence at the 3′ UTR of *RCC2* mRNA suggested that miR-1247 directly targets the 3′ UTR of *RCC2* mRNA. *RCC2*, also known as *TD-60*, is reportedly a component of the chromosomal passenger complex, which is a crucial regulator of chromosomes, the cytoskeleton, and membrane dynamics throughout mitosis [24, 25]. Additionally, *RCC2* knockdown induced cell cycle arrest at prometaphase in Hela cells due to failure of spindle assembly, suggesting that *RCC2* regulates cell cycle progression by modulating chromosome segregation and cell cleavage [26]. Recently, miR-29 was reported to regulate gastric cancer cell proliferation by targeting *RCC2* [27]. However, the contribution of *RCC2* to cell cycle regulation signaling is still poorly understood, and further studies in human cancers will be needed to clarify this issue.

In summary, our results indicate that aberrant expression of miR-1247 is regulated by DNA hypermethylation in pancreatic cancer. Functional analysis suggested that miR-1247 suppresses pancreatic tumorigenesis by targeting *RCC2* both *in vitro* and *in vivo*. Overall, all of these data suggest that miR-1247 might play a role as tumor suppressor in pancreatic cancer.

## MATERIALS AND METHODS

### Cell culture and 5-aza-dC treatment

Five human pancreatic cancer cell lines (AsPC-1, CFPAC-1, MIA PaCa-2, PANC-1, and SUIT-2) were used in this study. AsPC-1, CFPAC-1, MIA PaCa-2, and PANC-1 were obtained from the American Type Culture Collection (ATCC, Manassas, VA, USA). SUIT-2 cells were purchased from the Japanese Collection of Research Bioresources (JCBR, Japan) cell bank (The National Institute of Biomedical Innovation, Health and Nutrition (NIBIOHN), Japan). Cell line AsPC-1 was cultured in RPMI-1640 (Welgene, Daegu, Korea); CFPAC-1 cells were maintained in Iscove's Modified Dulbecco's Medium (IMDM, Welgene); and MIA PaCa-2, PANC-1, and SUIT-2 cells were grown in Dulbecco's Modified Eagle Medium (DMEM, Welgene). All cell culture medium was supplemented with 10% fetal bovine serum (Hyclone, Logan, UT, USA) and 1% antibiotic-antimycotic (Gibco, Grand Island, NY, USA). All cell lines were incubated at 37°C in atmospheric conditions of 20% O_2_ and 5% CO_2_. To investigate the effect of 5-aza-dC treatment, cells were treated with 5 μM 5-aza-dC (Sigma, St. Louis, MO, USA) for 72 h.

### Tissue samples

Pancreatic tumor tissues were collected from five patients, fixed in formalin, and embedded in paraffin. These samples were from patients who underwent primary surgical resection at the Johns Hopkins Hospital and were analyzed by bisulfite sequencing. All materials derived from the Johns Hopkins Hospital were obtained (with written informed consent) under institutional review board (IRB)-approved protocols. Total RNA and genomic DNA of normal pancreatic tissue samples were obtained from five normal pancreatic tissue donors (BioChain Institute, Newark, CA, USA).

### DNA methylation analysis: bisulfite sequencing and quantitative methylation-specific PCR (qMSP)

For methylation analysis, DNA was extracted using a standard phenol-chloroform method. A total of 2 μg of DNA was modified by bisulfite with the EZ DNA Methylation Kit™ (Zymo Research, Orange, CA, USA), which guarantees a > 99% conversion rate (of nonmethylated C nucleotides to U; protection of methylated cytosine residues). Methylation analysis of gene promoters was performed using MSP primer pairs located close to the putative transcription start site in the 5′ CpG island with 2 μl of bisulfite-treated DNA as template and JumpStart RedTaq DNA Polymerase (Sigma) for amplification as previously described [28]. For bisulfite sequence analysis, PCR amplicons were separated by 2% agarose gel electrophoresis, purified with the Gel Extraction Kit (Qiagen GmbH, Hilden, Germany), and cloned using the TOPO TA vector system (Invitrogen, Carlsbad, CA, USA). Individual clones were isolated and purified using NucleoSpin Plasmid Isolation Kit (Macherey-Nagel, Düren, Germany). Randomly selected positive clones (10–15 from each sample) were sequenced using the M13F primer, and methylation status of each CpG dinucleotide was analyzed. For quantification of methylation level of the RCC2 gene, qMSP amplification was performed on bisulfite-treated samples and normalized based on *Alu* element amplification. All methylation- and nonmethylation-specific primers were designed using MethPrimer (http://www.urogene.org/cgi-bin/methprimer/methprimer.cgi). All primer sequences are listed in Supplementary Table 1. qRT-PCR was performed using a CFX96^TM^ real-time system (BioRad, Hercules, CA, USA).

### Quantitative RT-PCR

Total RNA was isolated from human pancreatic cancer cell lines using TRI Solution (Bioscience Technology, Rockaway, NJ, USA) following the manufacturer's protocol. RNA was quantified using a NanoDrop 2000/2000c instrument (Thermo Scientific, Rockford, IL, USA), and 1 μg RNA was reverse-transcribed to cDNA using the iScript cDNA Synthesis Kit (BioRad). qRT-PCR was performed on a C1000 Thermal Cycler (BioRad) using the PCR primers listed in Supplementary Table 1.

### miRNA transfection

To ectopically express miR-1247 in pancreatic cancer cell lines, cells were transfected with 20 nM hsa-miR-1247 miScript mimic (MSY0005899, Qiagen), or Allstars Negative Control siRNA (1027281, Qiagen) using Lipofectamine 2000 (Invitrogen) following the manufacturer's instructions.

### Cell proliferation and viability assays

Cell proliferation was analyzed using the MTT assay. At 24 h after transfection of miRNA mimics or negative control, pancreatic cancer cells (2 × 10^5^ cells/well) were replated in 6-well plates and incubated at 37°C. After 72 h, cells were washed twice with phosphate-buffered saline (PBS), and 5 mg/ml MTT in PBS was added to each well followed by incubation for 4 h. After removing the MTT solution, a solubilization solution (dimethyl sulfoxide [DMSO]/ethanol:1:1) was added to each well to dissolve the formazan crystals. The absorbance at 570 nm was measured using a Paradigm^TM^ microplate reader (Beckman Coulter, Fullerton, CA, USA). At 48 h and 72 h after replating (5 × 10^4^ cells/well) in 6-well plates, cells were counted.

### Wound-healing assay

Cells were plated overnight to achieve a subconfluent monolayer in 6-well plates. Then, a scratch was made in the monolayer with a sterile 200 μl pipette tip, and cultures were washed twice with serum-free medium to remove floating cells. Cells were incubated in culture medium. After 16 h, wound healing was visualized by comparing photographs using a QImaging QI Click Camera system mounted on a phase-contrast Nikon microscope TS100 (Nikon, Melville, NY, USA). The distance traveled by the cells was determined by measuring the wound width at 16 h and subtracting it from the wound width at time 0.

### Migration and invasion assay

Cell migration was determined using transwell plates (24-well, 8 μm pore size, Corning Costar, NY, USA), and the invasion assay was carried using a Matrigel-coated invasion chamber (24-well, 8 μm pore size, Corning Costar). The upper chamber contained pancreatic cancer cells in Ham's F-12 (Welgene) supplemented with 1% FBS, and the lower chamber contained Ham's F-12 supplemented with 10% FBS. Cells were incubated for 16 h at 37°C in atmospheric conditions of 20% O_2_ and 5% CO_2_. Nonmigrated cells were scraped off the upper membrane with a cotton swab. Migrated cells remaining on the bottom membrane were counted after staining with Giemsa (Sigma). Photographs were taken using a QIcam image camera system mounted on Nikon ECLIPSE 80i microscope (Nikon).

### Western blot analysis

Total cell lysates (20 μg) were separated by SDS-PAGE and transferred to PVDF membranes (GE Healthcare Life Sciences, Piscataway, NJ, USA). The membranes were blocked with 5% skim milk dissolved in Tris-buffered saline (TBS) containing 0.02% Tween 20, and incubated overnight at 4°C with specific primary antibodies. The following primary antibodies were used: anti-RCC2 (Abcam, Cambridge, UK) and anti-β-actin (Sigma). The membranes were subsequently incubated with horseradish-peroxidase-conjugated secondary antibodies. Protein bands were visualized using a Fusion FX5 system (Vilber Lourmat, Eberhardzell, Germany).

### Dual-luciferase reporter assay

The 3′ UTR of *RCC2* mRNA was amplified and cloned into the firefly psiCHECK2 luciferase plasmid (Promega, Madison, WI, USA) at the *Xho*1/*Not*I restriction enzyme cleavage site to generate wild-type (wt) *RCC2* luciferase vector. Pancreatic cancer cells were co-transfected with wt *RCC2* or non-targeting negative control, and miR-1247 mimics together with *Renilla* psiCHECK2 vector for 48 h. Relative firefly/*Renilla* luciferase activity was then quantified using a Dual-Luciferase Reporter Assay (Promega) according to the manufacturer's protocol.

### Tumor xenograft assay

MIA PaCa-2 and PANC-1 cells were transfected with miR-1247 mimics or non-targeting negative controls for 48 h. Cells (1 × 10^6^) were then injected subcutaneously into the right flanks of 6 week old female nude mice. Five weeks after initial injection, mice were sacrificed, and the pancreatic cancer xenograft was processed for paraffin embedding and sectioning, followed by immunohistochemical analysis. MIA PaCa-2 and PANC-1 cells (1 × 10^6^) were transfected with miR-1247 mimics or non-targeting negative control according to the manufacturer's protocols, screened for stability, and then subcutaneously injected into the flank region. Tumor volume (V) was monitored by measuring the length (L) and width (W) of the tumor with calipers and was calculated with the formula V = (L×W^2^) × 0.5. Mice were sacrificed after 5 weeks and the tumors were removed for immunohistochemical analysis. Animal experiments were approved by the Institutional Animal Care and Use Committees of DIRAMS (DI-2016-018).

### Immunohistochemistry

Mouse tumor tissues were fixed with 10% formalin for 24 h, embedded in paraffin, and cut into 3 μm thick sections. Briefly, all paraffin sections were baked for 2 h at 65°C. Sections were deparaffinized with xylene and rehydrated in graded ethanol to distilled water. Sections were submerged in EDTA antigen-retrieval buffer (pH 8.0) and subjected to high-pressure treatment. After treatment with 0.3% H_2_O_2_ for 15 min to block endogenous peroxidase, the sections were treated with 1% BSA for 30 min to reduce nonspecific binding, and then sections were incubated with mouse monoclonal anti-RCC2 antibody (Abcam) overnight at 4°C. After washing, the sections were incubated with secondary antibody conjugated to a peroxidase-labeled polymer for 30 min; for color reactions, diaminobenzidine was used. For negative controls, the antibody was replaced with normal goat serum. All slides were counterstained with hematoxylin and photographed under a Nikon ECLIPSE 80i optical microscope (Nikon).

### TCGA data analysis

Public microRNA-sequencing (miRseq) and DNA methylation data performed in pancreatic adenocarcinoma (PAAD) were downloaded from the cancer genome atlas (TCGA, http://firebrowse.org/). Pearson's correlation coefficient was used to determine the correlation between the methylation level (beta value) of miR-1247 locus and the expression level (RPM, reads per million) of miR-1247 in 177 tumor samples.

### Statistical analyses

All experimental data are reported as means; error bars represent standard deviation (SD). Comparisons between values were performed using unpaired two-tailed Student's *t*-test. All statistical analyses were performed using Graphpad Prism 7.0 and the *p*-values < 0.05 considered significant.

## SUPPLEMENTARY MATERIALS FIGURES AND TABLES



## References

[1] Paulson AS, Tran Cao HS, Tempero MA, Lowy AM (2013). Therapeutic Advances in Pancreatic Cancer. Gastroenterology.

[2] Siegel RL, Miller KD, Jemal A (2016). Cancer statistics, 2016. CA Cancer J Clin.

[3] Bartel DP (2004). MicroRNAs: Genomics, Biogenesis, Mechanism, and Function. Cell.

[4] He L, Hannon GJ (2004). MicroRNAs: small RNAs with a big role in gene regulation. Nat Rev Genet.

[5] Lu J, Getz G, Miska EA, Alvarez-Saavedra E, Lamb J, Peck D, Sweet-Cordero A, Ebert BL, Mak RH, Ferrando AA, Downing JR, Jacks T, Horvitz HR (2005). MicroRNA expression profiles classify human cancers. Nature.

[6] Calin GA, Dumitru CD, Shimizu M, Bichi R, Zupo S, Noch E, Aldler H, Rattan S, Keating M, Rai K, Rassenti L, Kipps T, Negrini M (2002). Frequent deletions and down-regulation of micro- RNA genes miR15 and miR16 at 13q14 in chronic lymphocytic leukemia. Proceedings of the National Academy of Sciences.

[7] Michael MZ, Connor SM, van Holst Pellekaan NG, Young GP, James RJ (2003). Reduced accumulation of specific microRNAs in colorectal neoplasia. Molecular Cancer Research.

[8] Farazi TA, Spitzer JI, Morozov P, Tuschl T (2011). miRNAs in human cancer. The Journal of Pathology.

[9] Takamizawa J, Konishi H, Yanagisawa K, Tomida S, Osada H, Endoh H, Harano T, Yatabe Y, Nagino M, Nimura Y, Mitsudomi T, Takahashi T (2004). Reduced expression of the let-7 microRNAs in human lung cancers in association with shortened postoperative survival. Cancer Research.

[10] Suzuki H, Maruyama R, Yamamoto E, Kai M (2012). DNA methylation and microRNA dysregulation in cancer. Molecular Oncology.

[11] Toyota M, Suzuki H, Sasaki Y, Maruyama R, Imai K, Shinomura Y, Tokino T (2008). Epigenetic silencing of microRNA-34b/c and B-cell translocation gene 4 is associated with CpG island methylation in colorectal cancer. Cancer Research.

[12] Suzuki H, Takatsuka S, Akashi H, Yamamoto E, Nojima M, Maruyama R, Kai M, Yamano H-o, Sasaki Y, Tokino T, Shinomura Y, Imai K, Toyota M (2011). Genome-wide Profiling of Chromatin Signatures Reveals Epigenetic Regulation of MicroRNA Genes in Colorectal Cancer. Cancer Research.

[13] Jones PA, Baylin SB (2007). The Epigenomics of Cancer. Cell.

[14] Lujambio A, Ropero S, Ballestar E, Fraga MF, Cerrato C, Setién F, Casado S, Suarez-Gauthier A, Sanchez-Cespedes M, Gitt A, Spiteri I, Das PP, Caldas C (2007). Genetic Unmasking of an Epigenetically Silenced microRNA in Human Cancer Cells. Cancer Research.

[15] Lujambio A, Calin GA, Villanueva A, Ropero S, Sánchez-Céspedes M, Blanco D, Montuenga LM, Rossi S, Nicoloso MS, Faller WJ, Gallagher WM, Eccles SA, Croce CM (2008). A microRNA DNA methylation signature for human cancer metastasis. Proceedings of the National Academy of Sciences.

[16] Yan H, Choi A-j, Lee BH, Ting AH (2011). Identification and Functional Analysis of Epigenetically Silenced MicroRNAs in Colorectal Cancer Cells. PLoS ONE.

[17] Kim J-G, Kim T-O, Bae J-H, Shim J-W, Kang MJ, Yang K, Ting AH, Yi JM (2014). Epigenetically regulated MIR941 and MIR1247 target gastric cancer cell growth and migration. Epigenetics.

[18] Calin GA, Croce CM (2006). MicroRNA signatures in human cancers. Nat Rev Cancer.

[19] Saito Y, Liang G, Egger G, Friedman JM, Chuang JC, Coetzee GA, Jones PA (2006). Specific activation of microRNA-127 with downregulation of the proto-oncogene BCL6 by chromatin-modifying drugs in human cancer cells. Cancer Cell.

[20] Bueno MJ, Pérez de Castro I, Gómez de Cedrón M, Santos J, Calin GA, Cigudosa Juan C, Croce CM, Fernández-Piqueras J, Malumbres M (2008). Genetic and Epigenetic Silencing of MicroRNA-203 Enhances ABL1 and BCR-ABL1 Oncogene Expression. Cancer Cell.

[21] Nadal E, Chen G, Gallegos M, Lin L, Ferrer-Torres D, Truini A, Wang Z, Lin J, Reddy RM, Llatjos R, Escobar I, Moya J, Chang AC (2013). Epigenetic Inactivation of microRNA-34b/c Predicts Poor Disease-Free Survival in Early-Stage Lung Adenocarcinoma. Clinical Cancer Research.

[22] Suzuki H, Yamamoto E, Nojima M, Kai M, Yamano H-o, Yoshikawa K, Kimura T, Kudo T, Harada E, Sugai T, Takamaru H, Niinuma T, Maruyama R (2010). Methylation-associated silencing of microRNA-34b/c in gastric cancer and its involvement in an epigenetic field defect. Carcinogenesis.

[23] Bommer GT, Gerin I, Feng Y, Kaczorowski AJ, Kuick R, Love RE, Zhai Y, Giordano TJ, Qin ZS, Moore BB, MacDougald OA, Cho KR, Fearon ER (2007). p53-Mediated Activation of miRNA34 Candidate Tumor-Suppressor Genes. Current Biology.

[24] Martineau-Thuillier S, Andreassen RP, Margolis LR (1998). Colocalization of TD-60 and INCENP throughout G2 and mitosis: evidence for their possible interaction in signalling cytokinesis. Chromosoma.

[25] Skoufias DA, Mollinari C, Lacroix FB, Margolis RL (2000). Human Survivin Is a Kinetochore-Associated Passenger Protein. The Journal of Cell Biology.

[26] Mollinari C, Reynaud C, Martineau-Thuillier S, Monier S, Kieffer S, Garin J, Andreassen PR, Boulet A, Goud B, Kleman J-P, Margolis RL (2003). The Mammalian Passenger Protein TD-60 Is an RCC1 Family Member with an Essential Role in Prometaphase to Metaphase Progression. Developmental Cell.

[27] Matsuo M, Nakada C, Tsukamoto Y, Noguchi T, Uchida T, Hijiya N, Matsuura K, Moriyama M (2013). MiR-29c is downregulated in gastric carcinomas and regulates cell proliferation by targeting RCC2. Molecular Cancer.

[28] Herman JG, Graff JR, Myohanen S, Nelkin BD, Baylin SB (1996). Methylation-specific PCR: a novel PCR assay for methylation status of CpG islands. Proc Natl Acad Sci USA.

