# Supplementary material for: Epigenetically altered miR-1247 functions as a tumor suppressor in pancreatic cancer
Source: Oncotarget. 2017 Feb 24;8(16):26600–12. doi: 10.18632/oncotarget.15722 (PMC5432282; doi:10.18632/oncotarget.15722)
Supplement: Supplementary file 1 [file oncotarget-08-26600-s001.pdf]

# Epigenetically altered miR-1247 functions as a tumor suppressor in pancreatic cancer

## Supplementary Materials

### Supplementary Table 1: PCR primer information in this study

| Representative miRNA | Experiment | | Primer sequences (5′–3′) | |
|---|---|---|---|---|
| | | | Forward | Reverse |
| MiR1247 | Primary expression | | AACGCTCAGCACCCATTTAC | CGGACGTTGCTCTCTACCC |
| | Mature expression | | ACCCGTCCCGTTCGTCCCCGGA | CTGTGAATGCTGCGACTACGAT |
| | BS-seq | | TTAGTAGGGAGTAGGGTAGGGTGTT | ATTAAAAACAAACAAAAAAAATCAC |
| | MSP | Unmethylation | GTGGGGTGGTGTTGGTTTTG | AAATACACCCAACAACCAAAACTACACTAAACA |
| | | Methylation | GGGGATTTTAGGTTTAGATTTAATTGGCGTTC | TCAAAAATCCCACCTAATAACCCCG |
| ACTB | Normalization | | CATCCACGAAACTACCTTCAACTCC | GAGCCGCCGATCCACACG |
| 55 | Normalization | | TGGGAATACCGGGTGCTGT | CTGTGAATGCTGCGACTACGAT |
| Alu | Normalization | | ATTAGTCGGGCGTGGTGG | CCCGAATTCAAACGATTCTCC |

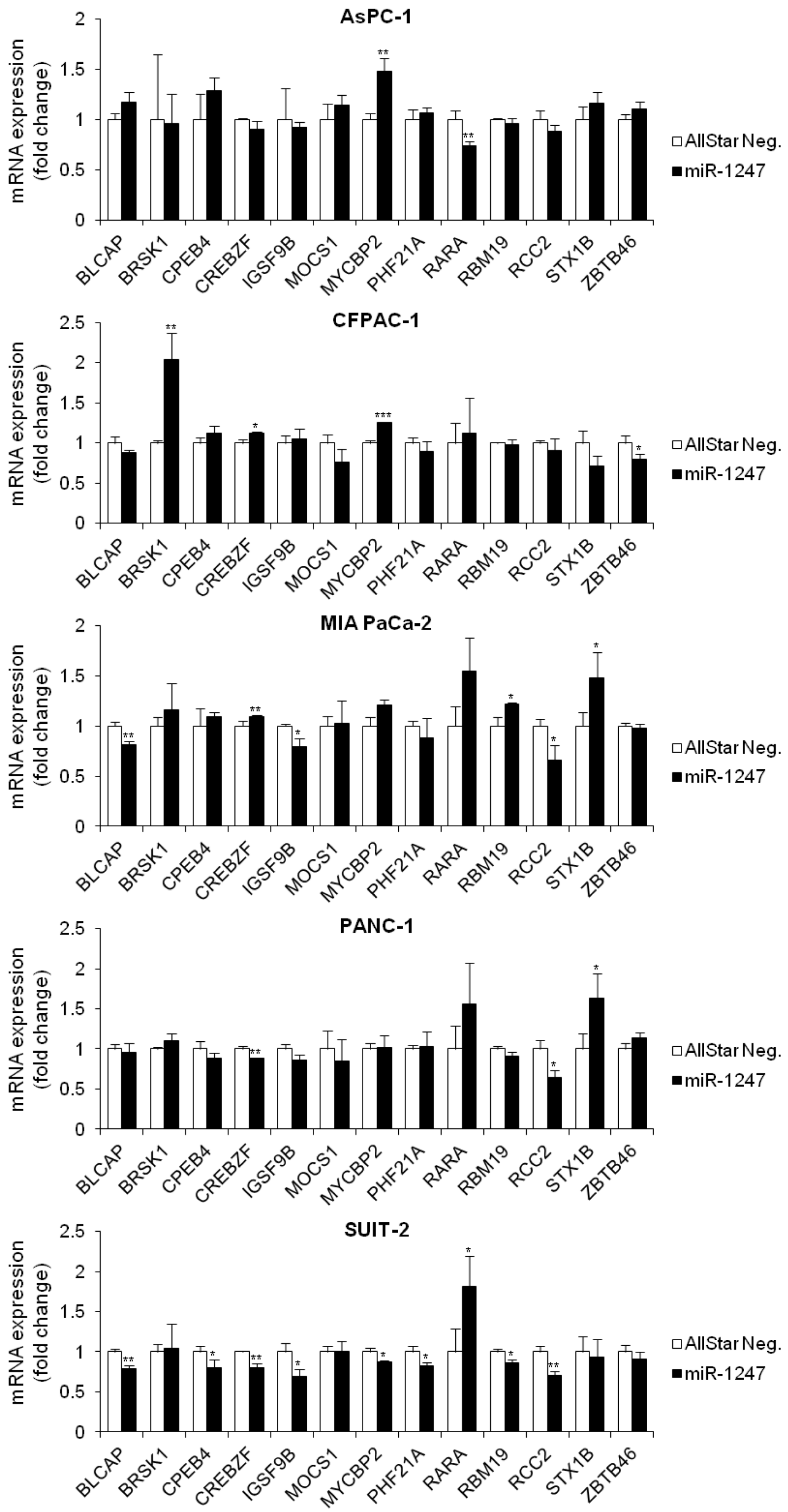


**Supplementary Figure 1: Putative candidate target genes of miR-1247 in pancreatic cancer cell lines.** qRT-PCR was carried out in 5 different pancreatic cancer cells transfected with non-targeting negative control (All Star neg.) and miR-1247 mimics with target genes. *indicates statistically significance decrease of gene expression level compared to control (*$P < 0.05$, **$P < 0.01$, ***$P < 0.001$).
